# Eye Tracking as Biomarker Compared to Neuropsychological Tests in Parkinson Syndromes: An Exploratory Pilot Study Before and After Deep Transcranial Magnetic Stimulation

**DOI:** 10.3390/brainsci15020180

**Published:** 2025-02-11

**Authors:** Celine Cont, Nathalie Stute, Anastasia Galli, Christina Schulte, Lars Wojtecki

**Affiliations:** 1 Department for Neurology and Neurorehabilitation, Hospital Zum Heiligen Geist, Academic Teaching Hospital of the Heinrich-Heine-University Duesseldorf, 47906 Kempen, Germany; celine.cont@artemed.de (C.C.);; 2 Institute of Clinical Neuroscience and Medical Psychology, Medical Faculty, Heinrich-Heine-University Düsseldorf, 40225 Düsseldorf, Germany

**Keywords:** Parkinson’s disease, eye tracking, deep transcranial magnetic stimulation, biomarker, neuromodulation

## Abstract

**Background/Objectives:** Neurodegenerative diseases such as Parkinson’s disease (PD) are becoming increasingly prevalent, necessitating diverse treatment options to manage symptoms. The effectiveness of these treatments depends on accurate and sensitive diagnostic methods. This exploratory pilot study explores the use of eye tracking and compares it to neuropsychological tests on patients treated with deep transcranial magnetic stimulation (dTMS). **Methods**: We used the HTC Vive Pro Eye VR headset with Tobii eye tracker to measure eye movements in 10 Parkinson syndrome patients while viewing three 360-degree scenes. Eye movements were recorded pre- and post-dTMS, focusing on Fixation Duration, Longest Fixation Period, Saccade Rate, and Total Fixations. Neuropsychological assessments (MoCA, TUG, BDI) were conducted before and after stimulation. dTMS was performed using the Brainsway device with the H5 helmet, targeting the motor cortex (1 Hz) and the prefrontal cortex (10 Hz) for 7–12 sessions. **Results:** ROC analysis indicated a moderate ability to differentiate between states using eye movement parameters. Significant correlations were found between changes in the longest fixation period and MoCA scores (r = 0.65, *p* = 0.025), and between fixation durations and BDI scores (r = −0.55, *p* = 0.043). Paired *t*-tests showed no significant differences in eye movement parameters, but BDI scores significantly reduced post-dTMS (t(5) = 2.57, *p* = 0.049). **Conclusions**: Eye-tracking parameters, particularly the Longest Fixation Duration and Saccade Rate, could serve as sensitive and feasible biomarkers for cognitive changes in Parkinson’s Syndrome, offering a quick alternative to traditional methods. Traditional neuropsychological tests showed a significant improvement in depressive symptoms after dTMS. Further research with larger sample sizes is necessary to validate these findings and explore the diagnostic utility of eye tracking.

## 1. Introduction

Neurodegenerative diseases such as Parkinson’s disease are becoming increasingly prevalent, necessitating a range of treatment options to manage their symptoms [1]. However, the effectiveness of these treatments hinges on the availability of accurate and sensitive diagnostic methods to measure their impact. This pilot study focuses on the comparison of neuropsychological assessments and eye-tracking metrics rather than evaluating the effects of dTMS. By analyzing measurements before and after dTMS, the study aims to assess the feasibility and sensitivity of eye tracking as a potential diagnostic tool. Parkinson’s disease (PD) is a progressive neurodegenerative disorder characterized by a range of motor symptoms, including bradykinesia, rigidity, tremor, and postural instability, as well as non-motor symptoms such as cognitive impairment, mood disorders, and autonomic dysfunction [2]. Traditional therapeutic approaches include pharmacological treatments, primarily using dopaminergic agents, and surgical interventions such as deep brain stimulation (DBS) [3,4]. However, these treatments often have limitations and side effects, prompting the exploration of non-invasive neuromodulation techniques such as transcranial magnetic stimulation (TMS) [5,6].

One specific type of TMS is deep transcranial magnet stimulation (dTMS), which involves using magnetic fields to stimulate deeper brain structures and has shown promise in various neurological and psychiatric disorders, including PD. A study by Shirota et al. [7] demonstrated that dTMS could modulate cortical excitability and improve motor symptoms in PD patients. Another study by Fregni et al. [6] indicated that dTMS targeting the motor cortex could lead to significant improvements in motor performance and quality of life. Recently, we published the first real-world German data and demonstrated that drTMS is a safe intervention for managing resistant Parkinson symptoms [8]. Moreover, we found that drTMS had a positive impact on depressive symptoms, indicating a potential benefit for mood improvement in patients undergoing this treatment. Low-frequency transcranial magnetic stimulation (TMS) at 1 Hz targeting the motor cortex has been studied for its potential to improve motor symptoms in Parkinson’s disease (PD). For instance, Lefaucheur et al. [9] found that low-frequency rTMS of the motor cortex could improve motor symptoms in PD patients by reducing cortical excitability. This was further supported by the study from Pal et al. [10], which showed significant improvements in motor performance following low-frequency stimulation. High-frequency TMS at 10 Hz targeting the prefrontal cortex has shown potential benefits in treating non-motor symptoms of PD, such as depression and cognitive impairment. For example, Benninger et al. [11] conducted a randomized controlled trial demonstrating that high-frequency rTMS over the prefrontal cortex significantly improved mood symptoms in PD patients. Similarly, studies [7,12] have shown that high-frequency TMS can enhance cognitive functions and executive control in PD patients, providing evidence of its efficacy in treating non-motor symptoms.

Eye tracking provides objective and quantitative measurements of eye movements, making it a sensitive tool for detecting subtle oculomotor abnormalities in neurodegenerative diseases, such as PD. Studies have shown that eye tracking can reveal early signs of cognitive and motor impairments, which might not be detected through traditional clinical assessments. Multiple brain regions, including the fronto-insular cortex, anterior cingulate cortex, supplementary motor area, superior colliculi, and thalamus, have been shown to be activated during fixation tasks [13]. Additionally, the bilateral dorsolateral prefrontal cortex is associated with fixation durations [14]. Executive function engages various cortical and subcortical areas, which are involved in tasks such as saccades, visual searching, and social cognition tasks. Therefore, eye tracking can be used to measure the bridge between behavior, brain function, and neural mechanisms. Ansons et al. [15] demonstrated that eye tracking could detect early cognitive decline in PD patients, while Brien et al. [16] showed that it could identify state and classification of cognitive and motor dysfunction in PD. Eye tracking has emerged as a valuable tool for assessing oculomotor function in PD. Studies have shown that PD patients exhibit various eye movement abnormalities, including increased fixation duration, reduced saccade frequency, and impaired smooth pursuit. Uc et al. [17] reported that PD patients had longer fixation durations and fewer saccades compared to healthy controls. Similarly, Mosimann et al. [18] found that PD patients demonstrated impaired smooth pursuit and increased latency in saccades.

Combining TMS and eye tracking offers a novel approach to understanding the neural mechanisms underlying oculomotor dysfunction in PD. A study by Mano et al. [19] indicated that rTMS could modulate eye movement control and improve oculomotor performance in PD patients. These findings suggest that TMS, particularly when combined with eye tracking, could serve as an effective therapeutic and diagnostic tool in PD. Especially free exploration in eye tracking, where participants are allowed to view visual stimuli in a natural and unrestricted manner, is crucial as it mimics real-world visual behavior. Unlike controlled tasks or paper-pencil tests, free exploration captures spontaneous eye movements and provides insights into how patients interact with their environment. This approach is particularly relevant for PD patients, whose real-world visual and cognitive challenges might not be fully captured in structured test settings. Tatler et al. [20] have emphasized the importance of naturalistic eye movement recordings in understanding visual attention and cognitive processes.

The present study aimed to evaluate eye movement parameters and neuropsychological test scores among Parkinson’s Syndrome (PS) patients to detect the effectiveness of deep transcranial magnetic stimulation (dTMS). Here, we sought to determine whether eye movement parameters could serve as sensitive biomarkers for cognitive and emotional changes in this population in comparison to neuropsychological tests. The following hypotheses were tested:

(I) Eye tracking in virtual reality serves as a sensitive measurement for Parkinson’s syndrome.
(II) Changes in neuropsychological tests correlate with changes in eye tracking measurements.
(III) There is a significant difference in eye movements as well as neuropsychological tests before and after stimulation.

## 2. Materials and Methods

### 2.1. Participants

We analyzed data from 10 patients (8 males, 2 females) with various forms of Parkinson’s syndrome at the Hospital zum Heiligen Geist in Kempen, Germany (see Table 1). Exclusion criteria for receiving deep repetitive transcranial magnetic stimulation (drTMS) included diagnosed epilepsy, pregnancy, presence of an implanted pacemaker or other metal implants, and alcohol and/or drug use on the day before or on the day of stimulation. Inclusion criteria comprised (i) a diagnosis of Parkinson’s syndrome (PS) and (ii) refractory hypokinetic or tremor symptoms despite levodopa medication or the necessity to reduce levodopa dose due to side effects. The diagnostic criteria for PS included bradykinesia and at least one of the following: rest tremor, muscular rigidity, or disturbances of posture and gait. The primary treatment goal or symptom for each patient was established before stimulation. Patients were on various medication regimens, primarily involving levodopa and its combinations. Details are provided in Table 1.

### 2.2. Eye Tracking Measurements in Virtual Reality

For this study, we used the HTC VIVE Pro Eye VR headset (HTC Corporation, Taoyuan City, Taiwan), which features an integrated Tobii eye tracker (Technology Inc., Danderyd, Sweden) to measure participants’ eye movements. SR_Runtime was utilized to enable eye tracking. Two base stations were required to track the VR headset. The VR hardware operated with Steam VR version 1.14.16. The display was a dual OLED 3.5″ diagonal screen with a resolution of 1440 × 1600 pixels per eye (2880 × 1600 pixels combined) and a refresh rate of 90 Hz. The field of view was 110°, which also represents the trackable field for the eye tracking integrated into the HTC VIVE Pro Eye. Tobii Eye Tracking accuracy within FOX 20 ranged from 0.5° to 1.1°. Gaze origin and direction were recorded in a three-dimensional, right-handed coordinate system. The experiment was developed in Unity. Eye tracking data were preprocessed by the Tobii XR SDK (Tobii Technology Inc., Danderyd, Sweden), which provides a three-dimensional gaze direction vector in the right-handed coordinate system with normalized gaze data between −1 and 1. Time data were recorded with a timestamp in the SRanipal SDK, which also logged the frame sequence provided by HTC.

### 2.3. Procedure

**Figure 1:**
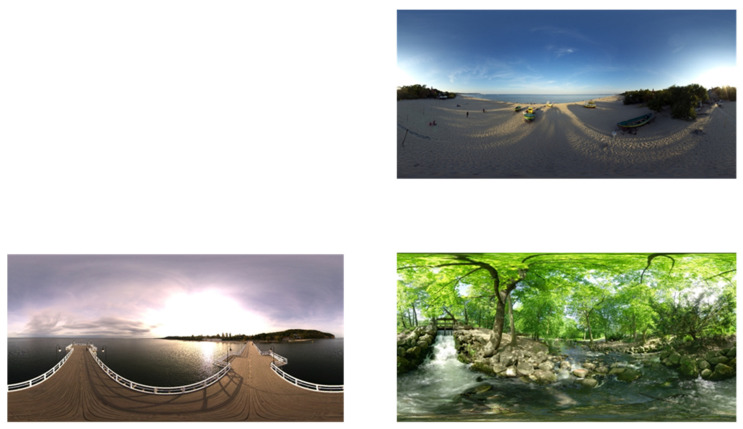
**Three scences in VR.** Symmetrical scence showing a beach, a pier, and a park scene with a river.

To create the experimental stimuli, an independent programmer developed three static 360-degree scenes, each representing a symmetrical environment: a beach, a pier, and a park scene with a river (see Figure 1). These scenes were selected to ensure a variety of visual stimuli while maintaining symmetrical properties. Participants viewed each scene for one minute, resulting in a total experiment duration of three minutes. Before viewing each scene, an eye-tracking calibration was conducted.

Eye movements were recorded before and right after the final dTMS session, focusing on the following parameters: Fixation Duration (ms), Longest Fixation Period (ms), Saccade Rate, and Total Number of Fixations. Fixations were defined as any eye movements where the velocity was less than 40 degrees per second. Eye movements exceeding this threshold were classified as saccades.

To prevent the possibility that changes in eye movements were an effect of the medication, care was taken to ensure that the pre- and post-testing occurred at approximately the same time of day (see Table 2). The Parkinson’s patients were admitted as inpatients, which ensured that they took their medication at the same time each day. On average, the time difference between the pre- and post-tests was −0.65 h (M = −0.65, excluding patient 5 since that patient does not take levodopa).

### 2.4. Neuropsychological Assessments

Moreover, neuropsychological tests were assessed before the first stimulation and after the last stimulation. To include cognitive, mood, and motor functions, the following tests were used:

The Montreal Cognitive Assessment (MoCA) was used to evaluate cognitive performance across multiple domains, including memory, attention, language, visuospatial abilities, and executive functions. The test consists of tasks such as word recall, clock drawing, and serial subtraction, with a total score ranging from 0 to 30 [21]. Scores below 26 were considered indicative of cognitive impairment. The MoCA is a validated tool widely used for detecting mild cognitive impairment and monitoring cognitive changes over time.

The Timed Up and Go (TUG) test was performed to assess functional mobility and balance. Participants began seated in a standard chair, stood up, walked 3 m at a comfortable pace, turned around, returned to the chair, and sat down. The time taken to complete the task was recorded in seconds. Higher times indicate reduced mobility and an increased risk of falls. The TUG is a simple and reliable measure frequently used in clinical and research settings to evaluate motor function [22].

The Beck Depression Inventory Fast Screen (BDI-II FS) is a brief, 7-item version of the full Beck Depression Inventory-II (BDI-II) designed to assess depressive symptoms, particularly in populations with medical or neurological conditions [23]. Unlike the full BDI-II, this version excludes somatic symptoms (e.g., fatigue, sleep disturbance, and appetite changes) that could overlap with physical symptoms of conditions like Parkinson’s disease. Each item evaluates cognitive and affective aspects of depression, such as sadness, pessimism, and loss of interest, using a 4-point Likert scale ranging from 0 (not at all) to 3 (severe). The total score ranges from 0 to 21, with higher scores indicating greater severity of depressive symptoms.

### 2.5. Deep Transcranial Magnetic Stimulation

For the deep transcranial magnetic stimulation (dTMS), we utilized the Brainsway (Brainsway Ltd., Jerusalem, Israel) dTMS device equipped with the H5 helmet, which is specifically designed for Parkinson’s disease indications. The Brainsway dTMS system features a stimulator, a touchscreen interface, and an efficient cooling system. The dTMS stimulation was conducted in two separate sessions: one targeting the motor cortex and the other targeting the prefrontal cortex. The patient wore a helmet containing the H5 coil and a measuring tape to precisely determine the motor threshold before the first session. The motor threshold was identified by gradually increasing the stimulation intensity until a visible muscle contraction was observed in the hand or thumb. During the first session, the motor cortex was stimulated with low-frequency dTMS at 1 Hz. In the second session, the prefrontal cortex was stimulated with high-frequency dTMS at 10 Hz. To minimize discomfort from the noise generated during the stimulation, patients were provided with earplugs. Each session was conducted under controlled conditions to ensure accurate targeting and patient safety. Patients were stimulated between 7 and 12 sessions in total every second day. Thus, most patients were stimulated for 4 weeks, 3 times a week.

## 3. Results

(I) Eye tracking in virtual reality serves as a sensitive measurement for Parkinson’s Syndrome [confirmed].

Receiver Operating Characteristic (ROC) analysis was conducted to evaluate the discriminative ability of various eye movement parameters. The results indicated a moderate ability to differentiate between states using these parameters. The area under the curve (AUC) for Mean Fixation Duration was 0.60 with a 95% confidence interval (CI) of [0.31, 0.89]. The AUC for Longest Fixation Period was 0.66, with a 95% CI of [0.37, 0.94]. For the Total Number of Fixations, the AUC was 0.54 with a 95% CI of [0.24, 0.84], and for the Saccade Rate, the AUC was 0.72 with a 95% CI of [0.46, 0.99]. Therefore, hypothesis I can be confirmed by showing a moderate ability to differentiate between states.

Effect sizes (Cohen’s d) were calculated to determine the magnitude of changes in eye movement parameters and neuropsychological test scores. The Cohen’s d for Mean Fixation Duration was 0.10, with a 95% CI of [−0.57, 0.76]; for Longest Fixation Period, it was 0.35, with a 95% CI of [−0.33, 1.01]; for the Total Number of Fixations, it was 0.20, with a 95% CI of [−0.46, 0.86]; and for the Saccade Rate, it was 0.45, with a 95% CI of [−0.23, 1.12].

(II) Changes in neuropsychological tests correlate with changes in eye tracking measurements [partly confirmed].

Pearson correlations were conducted to examine the relationships between changes in eye movement parameters and changes in neuropsychological test scores (see Table 3). A positive correlation was observed between changes in the Longest Fixation Period and improvements in MoCA scores, r(8) = 0.65, *p* = 0.025. Saccade Rate changes were negatively correlated with TUG test times, r(4) = −0.45, *p* = 0.082, though this was not statistically significant. Changes in Fixation Durations correlated negatively with BDI-II FS scores, r(7) = −0.55, *p* = 0.043. Additionally, a positive, non-significant correlation was observed between Mean Fixation Duration and MoCA scores, r(8) = 0.45, *p* = 0.187. A negative, non-significant correlation was found between Longest Fixation Period and TUG test times, r(4) = −0.40, *p* = 0.221, and between Total Fixations and BDI scores, r(7) = −0.39, *p* = 0.221. A positive, non-significant correlation was observed between Saccade Rate and MoCA scores, r(8) = 0.30, *p* = 0.421. A negative, non-significant correlation was found between Total Fixations and TUG test times, r(4) = −0.35, *p* = 0.312, and a very low, non-significant correlation was observed between Saccade Rate and BDI scores, r(7) = 0.10, *p* = 0.781. Therefore, hypothesis II can be confirmed for some parameters (see Table 3).

(III) There is a significant difference in eye movements [rejected] as well as neuropsychological tests [partly confirmed] before and after stimulation.

Paired *t*-tests were also conducted to compare pre- and post-dTMS measurements of neuropsychological test scores (see Table 4 for mean scores and Table 5 for paired *t*-test). For MoCA scores, there was no significant difference, t(9) = −1.27, *p* = 0.238. For TUG test times, there was no significant difference, t(4) = 0.37, *p* = 0.728. For BDI scores, there was a significant difference, t(5) = 2.57, *p* = 0.049, indicating a reduction in depressive symptoms. In sum, hypothesis III could not be confirmed based on the data collected.

Paired *t*-tests were conducted to compare pre- and post-dTMS measurements of eye movement parameters (see Table 5). For Mean Fixation Duration, there was no significant difference, t(9) = 0.053, *p* = 0.959. For Longest Fixation Period, there was no significant difference, t(9) = 1.019, *p* = 0.335. For the Total Number of Fixations, there was no significant difference, t(9) = 0.353, *p* = 0.732. For the Saccade Rate, there was no significant difference, t(9) = 0.960, *p* = 0.361.

## 4. Discussion

This exploratory pilot study aimed to evaluate the feasibility and sensitivity of eye tracking as a diagnostic tool compared to neuropsychological tests for detecting changes before and after dTMS in Parkinson’s Syndrome. By comparing these tools, we sought to establish a foundation for future research into eye tracking’s potential as an inclusive and efficient biomarker. For hypothesis I, if eye tracking serves as a sensitive measurement, analysis indicated a moderate ability to differentiate between states using eye movement parameters. Specifically, the area under the curve (AUC) values were 0.60 for Mean Fixation Duration, 0.66 for Longest Fixation Period, 0.54 for Total Number of Fixations, and 0.72 for Saccade Rate. Effect sizes (Cohen’s d) for these parameters were 0.10, 0.35, 0.20, and 0.45, respectively, indicating small to moderate changes. Therefore, hypothesis I can be confirmed.

For hypothesis II, if changes in neuropsychological tests correlate with changes in eye tracking measurements, significant correlations were found between changes in the Longest Fixation Period and MoCA scores (r = 0.65, *p* = 0.025), and between changes in Fixation Durations and BDI scores (r = −0.55, *p* = 0.043). Although the correlation between Saccade Rate changes and TUG test times was not statistically significant (r = −0.45, *p* = 0.082), it suggested a trend where higher Saccade Rates might be associated with better motor performance. Therefore, hypothesis II can be confirmed for some parameters.

For hypothesis III, if there is a significant difference in eye movement as well as in neuropsychological test scores, paired *t*-tests comparing pre- and post-dTMS measurements showed no significant differences in Mean Fixation Duration (t(9) = 0.053, *p* = 0.959), Longest Fixation Period (t(9) = 1.019, *p* = 0.335), Total Number of Fixations (t(9) = 0.353, *p* = 0.732), and Saccade Rate (t(9) = 0.960, *p* = 0.361). However, the BDI scores showed a significant reduction post-dTMS (t(5) = 2.57, *p* = 0.049), suggesting that dTMS may positively impact depressive symptoms in PD patients. In sum, the hypothesis III could not be confirmed based on the data collected.

The findings from this study contribute to evidence supporting the use of dTMS in PS treatment. The current study extends these findings by exploring the effects of dTMS on eye movement parameters and neuropsychological outcomes.

The significant correlation between changes in the Longest Fixation Period and MoCA scores aligns with earlier studies that have highlighted the relationship between eye movements and cognitive function in [24,25]. These findings suggest that eye movement parameters, particularly the Longest Fixation Period, could serve as reliable biomarkers for cognitive changes in PD. The moderate effect size observed for the Saccade Rate further underscores its potential utility as a diagnostic tool.

Eye tracking offers several advantages as a sensitive diagnostic tool. It provides objective, quantifiable data that can detect subtle changes in cognitive and emotional states, often before these are apparent in traditional neuropsychological tests [15]. Eye tracking can be conducted in a naturalistic setting, which may better reflect real-world functioning compared to paper-and-pencil tests. This is particularly important in PD, where cognitive and motor symptoms can fluctuate and be context-dependent. Parkinson’s patients often experience difficulties with verbal fluency or language in general, which can pose challenges for traditional neuropsychological tests. Eye tracking, by contrast, is entirely language-independent, making it better suited for such populations. However, neuropsychological tests also have advantages, such as not requiring specialized hardware or advanced technological setups. Furthermore, while eye tracking demands specific training to operate and interpret results, traditional tests are relatively straightforward to administer, requiring less technical expertise.

For the feasibility of using eye tracking, the study demonstrated that in just three minutes (duration of the eye tracking task), valuable insights about multiple cognitive domains could be provided, including attention, processing speed, and motor coordination. In comparison, traditional assessments such as the MoCA (which takes approximately 10–15 min), Timed Up and Go (about 5–10 min), and BDI (around 5–10 min), which were used for this study, required considerably more time per patient. Furthermore, the Timed Up and Go was not feasible for all participants, as some were wheelchair-bound and unable to walk. The eye-tracking method, however, enabled the assessment of both cognitive and motor functions while patients were seated, highlighting its inclusivity, efficiency, and potential for broader clinical application.

The reduction in BDI-II FS scores post-dTMS is consistent with prior research indicating the antidepressant effects of TMS [8,12]. This is particularly relevant for PD patients, who often experience comorbid depression [26]. The observed correlation between changes in BDI-II FS scores and Fixation Durations suggests that eye tracking parameters may reflect not only emotional states but also cognitive and attentional mechanisms relevant to social functioning. Future research could build on these findings by incorporating measures of social cognition and evaluating how changes in eye movement patterns relate to improvements in real-world social interactions. This could provide deeper insights into the broader implications of dTMS and eye tracking for enhancing quality of life in PD.

Although the paired *t*-tests did not show significant changes in most eye movement parameters, the observed trends and effect sizes highlight the need for further investigation with larger sample sizes. The non-significant negative correlation between Saccade Rate changes and TUG test times suggests that eye movements might be linked to motor performance, albeit weakly in this study. Future research should explore this relationship more deeply, considering the small sample size and variability in TUG test times. Neuropsychological tests like the MoCA, TUG, and BDI provide valuable insights into cognitive, motor, and emotional functions. The MoCA assesses various cognitive domains, while the TUG test measures mobility and balance, and the BDI evaluates depressive symptoms. However, these tests can be subjective and influenced by patient effort and external factors. In contrast, eye tracking offers quantitative and objective measurements that can detect subtle changes in oculomotor function. Eye tracking can be used to detect cognitive function. Cognitive psychology and eye-tracking research highlighted this by suggesting the *eye-mind assumption* [27]. This assumption suggests that there is a close temporal and spatial link between eye fixation and cognitive processing. The assumption demonstrates that eye movements provide information about cognitive processes. Therefore, eye-tracking measurements should be a key tool for future studies to investigate a new approach to detecting cognitive functions.

A significant limitation of this study is the small sample size, which may have reduced the statistical power to detect significant changes and correlations. The limited number of participants also increases the risk of type II errors, where meaningful differences or relationships could remain undetected. Furthermore, the lack of diversity within the sample, particularly in terms of age, gender, disease severity, and comorbidities, restricts the generalizability of the findings to broader populations. Parkinson’s disease is a heterogeneous condition, and the observed effects might not be representative of the wider population or specific subgroups, such as those with advanced disease stages or different treatment histories. Additional limitations include variability in TUG test performance, potentially influenced by differences in participant motivation or fatigue during testing, which could introduce noise into the data. Moreover, missing data for some neuropsychological test scores further complicates the interpretation of results, as these gaps may have biased the analysis. Another important limitation of this study is the absence of a control group for the drTMS treatment. Without a control group of participants without Parkinson’s syndrome or those who did not receive the intervention, it is challenging to determine whether the observed changes and correlations are truly attributable to the intervention or represent natural variability. A control group would provide a baseline for comparison, allowing researchers to isolate the specific effects of the intervention from other confounding factors, such as placebo effects, fluctuations in symptom severity, or environmental influences. Here, the two measurements were compared.

To address these issues, future studies should prioritize larger, more diverse cohorts that capture the demographic and clinical variability of Parkinson’s disease populations. Stratified sampling methods could be employed to ensure representation across disease stages, treatment modalities, and comorbid conditions.

Moreover, while this study focused on the effects of dTMS on eye movement parameters and neuropsychological outcomes, it did not explore the underlying neural mechanisms. Neuroimaging studies could provide valuable insights into how dTMS modulates brain activity related to eye movements and cognitive/emotional functions in PD.

## 5. Conclusions

In conclusion, this study provides preliminary evidence that eye movement parameters, particularly the Longest Fixation Period and Saccade Rate, hold potential as biomarkers for cognitive changes. Therefore, this study suggests the potential feasibility of eye tracking as a sensitive biomarker. Moreover, neuropsychological scores could show that dTMS may positively influence depressive symptoms in PD patients. The moderate effect sizes and significant correlations observed warrant further research with larger samples to validate these findings and fully elucidate the diagnostic utility of eye movements in PD. This exploratory pilot study suggests that eye tracking, due to its objective and sensitive nature, may offer a superior alternative to traditional neuropsychological tests in detecting subtle cognitive and emotional changes in PD.

## Figures and Tables

**Table 1:** Patient Diagnosis and Medication.

Patient	Diagnosis	Medication
1	PS ^1^	Levodopa
2	MSA ^2^	Levodopa
3	PSP ^3^	Levodopa
4	MSA ^2^	Levodopa
5	MSA ^2^	None
6	PD ^4^	Levodopa
7	Overlap Syndrome (CBD ^5^, PSP ^3^)	Levodopa
8	PD ^4^	Levodopa
9	PS ^1^	Levodopa
10	PS ^1^	Levodopa

Patient diagnoses and medication. ^1^ PS = atypical Parkinson syndrome, not further classified. ^2^ MSA = multiple system atrophy, ^3^ PSP = progressive supranuclear palsy, ^4^ PD = Parkinson’s disease, ^5^ CBD = corticobasal degeneration.

**Table 2:** Pre- and post-test time of eye-tracking parameters.

Patient	Pre-Test Time	Post-Test Time	Time Difference (Hours)
1	12:42	10:33	−2.15
2	13:09	11:35	−1.57
3	12:52	10:48	−2.07
4	13:58	11:51	−2.12
5	14:54	11:20	−3.57
6	12:43	12:05	−0.63
7	10:19	11:42	1.38
8	10:21	10:57	0.60
9	14:07	13:01	−1.10
10	11:26	13:17	1.85

**Table 3:** Pearson correlation coefficients between changes in eye movement parameters and changes in neuropsychological test scores. * Indicating a significant (*p* < 0.05) correlation.

Parameter	MoCA Change (r, *p*)	TUG Change (r, *p*)	BDI-II FS Change (r, *p*)
Longest Fixation Period Change	0.65, 0.025 *	−0.40, 0.221	−0.39, 0.221
Mean Fixation Duration Change	0.45, 0.187	-	−0.55, 0.043 *
Total Fixations Change	-	−0.35, 0.312	−0.39, 0.221
Saccade Rate Change	0.30, 0.421	−0.45, 0.082	0.10, 0.781

**Table 4:** Mean scores in neuropsychological test batteries administered before dTMS stimulation and after stimulation.

	M*pre*	M*post*
**Neuropsychological Tests**		
MoCA Scores	23.3	24.3
TUG Test Times	28.18	27.28
BDI Scores	3.67	1.67

**Table 5:** Paired *t*-test results for eye movement parameters and neuropsychological test scores pre- and post-dTMS. * indicates a significant (*p* < 0.05) value.

Parameter	t(df)	*p*-Value
**Eye Movement Parameters**		
Mean Fixation Duration	0.053 (9)	0.959
Longest Fixation Period	1.019 (9)	0.335
Total Number of Fixations	0.353 (9)	0.732
Saccade Rate	0.960 (9)	0.361
**Neuropsychological Tests**		
MoCA Scores	−1.27 (9)	0.238
TUG Test Times	0.37 (4)	0.728
BDI Scores	2.57 (5)	0.049 *

## Data Availability

The data are not publicly available due to containing information that could compromise the privacy of research participants.
